# Blood-Catalyzed Polymerization Creates Conductive Polymer in Live Zebrafish

**DOI:** 10.21203/rs.3.rs-3602290/v1

**Published:** 2024-06-25

**Authors:** Sanket Samal, Samantha Nelson, Zhiyi Du, Decheng Wang, Tianqi Wang, Chen Yang, Qing Deng, Elizabeth I. Parkinson, Jianguo Mei

**Affiliations:** 1 Department of Chemistry, Purdue University, West Lafayette, IN, USA.; 2 Department of Medicinal Chemistry and Molecular Pharmacology, Purdue University, West Lafayette, IN, USA.; 3 Department of Chemistry, Boston University, Boston, MA, USA.; 4 Department of Biological Sciences, Purdue University, West Lafayette, IN, USA.; 5 Purdue Institute for Inflammation, Immunology & Infectious Diseases, Purdue University, West Lafayette, IN, USA.; 6 Purdue University Center for Cancer Research, Purdue University, West Lafayette, IN, USA.

## Abstract

Conducting polymers are of great interest in bioimaging, bio-interfaces, and bioelectronics for their biocompatibility and the unique combination of optical, electrical, and mechanical properties. They are typically prepared outside through traditional organic synthesis and delivered into the biological systems. The ability to call for the polymerization ingredients available inside the living systems to generate conducting polymers *in vivo* will offer new venues in future biomedical applications. This study is the first report of *in vivo* synthesis of an n-doped conducting polymer (n-PBDF) within live zebrafish embryos, achieved through whole blood catalyzed polymerization of 3,7-dihydrobenzo[1,2-b:4,5-b′]difuran-2,6-dione (BDF). Prior to this, the efficacy of such a polymerization was rigorously established through a sequence of *in vitro* experiments involving Hemin, Hemoproteins (Hemoglobin, Myoglobin, and Cytochrome C), red blood cells, and the whole blood. Ultimately, *in cellulo* formed n-PBDF within cultured primary neurons demonstrated enhanced bio-interfaces and led to more effective light-induced neural activation than the prefabricated polymer. This underscores the potential advantages of synthesizing conducting polymers directly in living systems for biomedical applications.

## Introduction

Conducting polymers (CPs) have gained significant attention in biomedical applications due to their biocompatibility and tunable electronic, optical, and electrochemical properties.^1,2^ They are not naturally produced in living systems and are introduced externally for various applications such as bioimaging, drug delivery, biosensors, and neural interfaces.^3–7^ However, these strategies often result in poor bio-integration with soft tissues, creating a gap between *in vitro* and *in vivo* device performance and longevity.^8–10^ To overcome such shortcomings, attempts have been made to synthesize CPs within biological systems, with preliminary work showing that CPs can be safely synthesized *in vivo* with electrochemical polymerization.^11^ Recent work developed the *in vivo* assembly of CPs directly onto neural membranes, either by genetic engineering to express enzymes that catalyze polymerization^12–14^ or by using external oxidative enzymes that trigger endogenous metabolites (i.e., H_2_O_2_) to promote local polymerization in living fish and medicinal leeches.^15,16^ These methods still present limitations, such as the formation of toxic byproducts from over-expression of oxidative enzymes leading to cell apoptosis.^17^ Therefore, it is appealing to assemble CPs *in vivo* by only using endogenous metabolites to initiate and promote polymerization.

Recently, n-doped conducting polymer poly(3,7-dihydrobenzo[1,2-*b*:4,5-*b*’]difuran-2,6-dione) (n-PBDF) was reported, showing features such as high conductivity, and air/water stability, and biocompatibility.^18,19^ The reaction mechanism involves oxidative polymerization by mild oxidants and reductive doping by water, which could conceivably occur inside living organisms. Here, we discovered that utilizing endogenous enzymatic proteins, such as hemoproteins, can lead to efficacious *in vivo* polymerization of n-PBDF in aqueous media. We then demonstrated *in vivo* synthesis of n-PBDF through whole blood catalyzed oxidative polymerization and water-promoted reductive doping. We further verified that it is possible to form n-PBDF in live zebrafish embryos without any casualty, showing excellent biocompatibility. We eventually demonstrated the potential of *in cellulo* synthesized n-PBDF as photoacoustic transducers with enhanced bio-integration, enabling non-genetic neural stimulation in cultured primary neurons with a submillimeter resolution.

## Biocompatible Synthesis of n-PBDF in Aqueous Media

**Figure 1.**
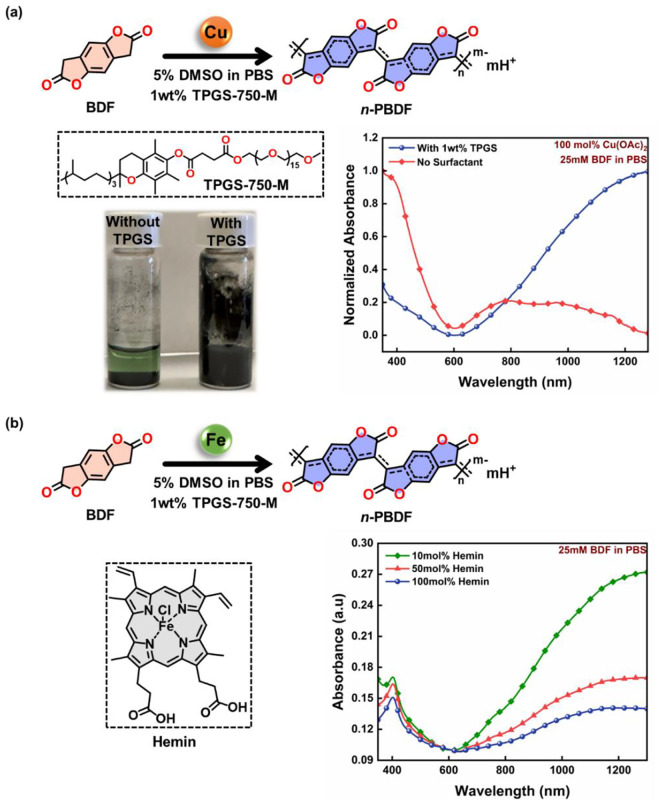
Biocompatible synthesis of n-PBDF in aqueous media at 37 °C. **a)** Copper facilitated polymerization in the presence/absence of TPGS-750-M as a surfactant, and **b)** Hemin promoted polymerization and the de-doping phenomenon.

Although the prior synthesis of n-PBDF is efficient, it is not suitable for biological systems. The BDF (3,7-dihydrobenzo[1,2-*b*:4,5-*b*’]difuran-2,6-dione) monomer has limited solubility in organic solvents and is insoluble in aqueous media. The previous synthesis methods necessitated high temperatures and employed dimethyl sulfoxide (DMSO) as a solvent. To surpass this limitation, we developed a method to synthesize n-PBDF in aqueous media using surfactants, similar to the synthesis of CP nanoparticles.^20^ One of the most promising options was found to be vitamin E-based TPGS-750-M (Tocopheryl Polyethylene Glycol Succinate).^21^ Using TPGS as a surfactant, n-PBDF can be polymerized in aqueous media through emulsion polymerization. By incorporating an insignificant amount (5%) of DMSO and 1% w/w TPGS, this method was found suitable for use in biological systems. (Figure 1)

To verify the effectiveness of the synthetic method, we first investigated the polymerization of n-PBDF in 1x phosphate buffered saline (PBS) (pH 7.4) media using copper acetate as the catalyst at body temperature (37 °C) with and without the surfactant. We found the polymerization method highly consistent, resulting in an aqueous n-PBDF ink that was analyzed using a UV-Vis-NIR spectrophotometer, showing strong absorption in the NIR region with the use of TPGS surfactant and matches the reported results (Figure 1a and Figure S2). In addition, TPGS-750-M’s effectiveness was compared to other surfactants, such as Triton X-100, which is widely used in biological systems for immunostaining and DNA extraction.^22,23^ The polymerization process was more efficient with TPGS-750-M than with Triton X-100, giving highly doped polymer using a smaller amount of surfactant (Figure S3). Although copper is highly competent as a catalyst, it is found in trace quantities in biological systems, and excessive levels of copper are toxic, making it unsuitable as a catalyst for *in vivo* polymerization applications. To increase the viability of the polymerization process, we became interested in using an endogenous iron-based catalyst, as iron is essential in biological systems and could facilitate redox reactions, making it the fundamental component for many bio-enzymes and proteins.^24^

## Endogenous Proteins Catalyzed Polymerization of n-PBDF

**Figure 2.**
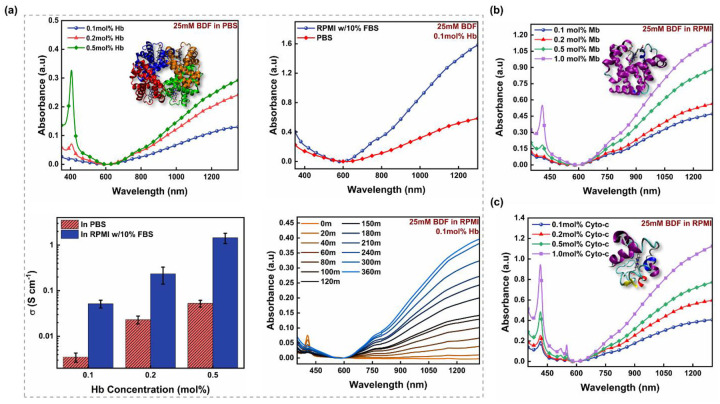
Hemoprotein catalyzed polymerization of n-PBDF in aqueous media at 37 °C. **a)** Hemoglobin (Hb) [Different concentrations of Hb (top left), Different aqueous media (top right), Conductivity of n-PBDF thin films (bottom left), and Polymerization progress at different time intervals (bottom right)], **b)** Myoglobin (Mb), and **c)** Cytochrome C (Cyto-c) (The results are representative of at least three unique experiments)

We evaluated iron’s ability to catalyze the efficient polymerization of n-PBDF by examining hemin as a potential source of iron. Hemin contains a ferric ion and a coordinating chloride ligand resembling the critical components of many hemoproteins. We found that the hemin catalyzed the polymerization of n-PBDF successfully. However, the polymer’s doping level decreases as the amount of hemin increases from 10 mol% to 100 mol% (Figure 1b), likely due to the free Fe^3+^ ion in the solution that de-dopes the polymer and results in it precipitating out from the n-PBDF polymer ink. However, this observation opened a potential pathway for using heme-containing bioactive proteins to facilitate *in vivo* polymerization of n-PBDF. Of all hemoproteins, hemoglobin (Hb) is the most widely recognized as it plays the crucial role of transporting oxygen in the vascular system of animals.^25^ With Hb as the catalyst, we noticed considerable polymerization with only 0.1 mol% of Hb, causing the solution to turn black within 20 minutes. By increasing the Hb amount from 0.1 mol% to 0.5 mol%, we noticed an improvement in the doping level of n-PBDF, along with a higher conversion rate (Figure 2a). In addition, when the reaction was performed under the oxygen environment, the catalyst’s reactivity increases, giving higher conversion and doping levels (Figure S7).

To mimic *in vivo* conditions, we switched from PBS to RPMI-1640 media solution containing 10% fetal bovine serum (FBS) and 1% Penicillin-Streptomycin. We observed that polymerization is more effectual in the RPMI media than in the PBS solution. We further conducted a comprehensive kinetic study using UV-Vis-NIR measurements to assess the effects of switching solvents from PBS to RPMI (Figure 2a and Figure S4). We observed that the reaction occurred faster in RPMI media compared to PBS while maintaining the same concentrations of BDF and Hb. The RPMI media with FBS most likely contains some Hb and other hemoproteins, leading to better conversion and higher doping levels within a six-hour reaction interval compared to PBS. The conductivity measurements of n-PBDF thin films were consistent with those of polymers synthesized in different mediums (Figure 2a). Regardless of the Hb concentration, we observed a notable rise in conductivity when changing the medium from PBS to RPMI, leading us to achieve a conductivity of 1.6 S cm^−1^. This level of conductivity is comparable to other conducting materials that are directly assembled in living systems^26^ and matches the conductivity requirements for various biomedical applications.^27,28^ It is noted that the presence of insulating hemoglobin and surfactant in the thin films might lead to an underestimation of conductivity.

To investigate the universal nature of the heme-containing catalytic system, we analyzed various other hemoproteins, such as myoglobin (Mb) and cytochrome C (Cyto-c) (Figure 2b & 2c). Through our screening, we noticed that regardless of the type of protein utilized, we observed comparable outcomes with increased conversion and higher doping levels when the quantity of protein was increased. It was observed that efficient polymerization of n-PBDF occurred when at least 0.1 mol% of protein was present in the reaction media (Figure 2). This amount is comparable to the quantity of Hb found in a single drop of blood from a healthy adult female. However, Hb is undeniably the fastest and most efficient because it possesses four heme groups, a distinct advantage over Mb and Cyto-c, which only contain one.

**Figure 3.**
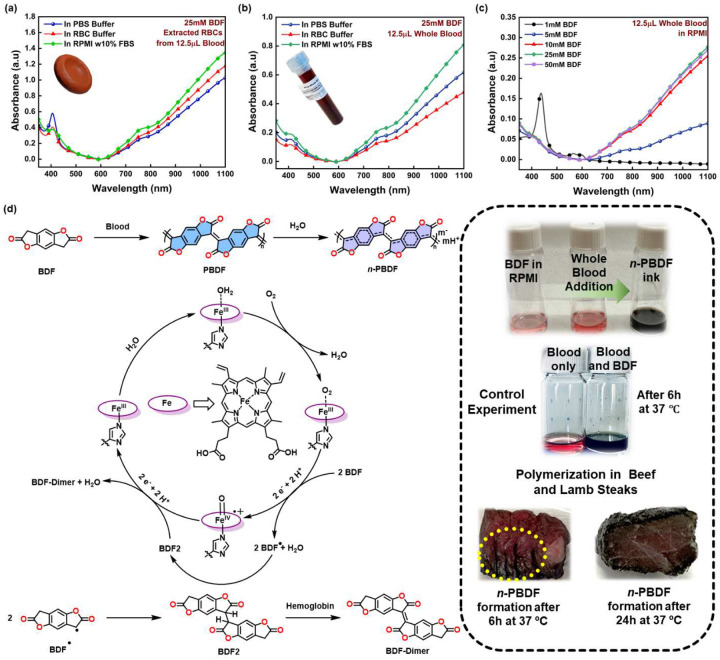
Red blood cells and whole blood catalyzed polymerization of n-PBDF in aqueous media at 37 °C. **a)** RBCs as the catalyst, **b)** Whole blood as the catalyst, **c)** Minimum BDF concentration needed for polymer formation in whole blood, and **d)** Proposed mechanism and catalytic cycle for forming n-PBDF (**inset**: control experiments using whole blood, and formation of n-PBDF in lamb and beef steaks with an injection of 100μL of 25mM BDF solution after incubation at 37 °C). (The results are representative of at least three unique experiments)

To delve deeper into the impact of BDF concentration on the polymerization, we altered the BDF concentration from 10 mM to 50 mM while maintaining a steady Hb concentration of 25 μM (Figure S5). We observed that irrespective of the BDF concentrations, the polymerization rate remains the same. For low BDF concentration of 10 mM, complete conversion was observed after 2 hours, while for higher BDF concentrations of 25 mM and 50 mM, the reaction continued for 6 hours and then gradually slowed down. This phenomenon can be attributed to the slow, gradual iron release from the heme core, leading to the decomposition of Hb. As a result, the intensity of the Hb peak (410 nm, specific to Fe^3+^-heme)^29^ decreases and eventually disappears after about six hours. Furthermore, to better comprehend the catalytic effect of Hb, we altered the Hb concentration from 0.1 mol% to 0.5 mol% while maintaining a constant BDF concentration of 25 mM (Figure S6). We observed that the polymerization rate increases as the concentration of the Hb increases. Hence, it can be inferred from our observations that the enzymatic polymerization of n-PBDF using hemoproteins follows zero-order enzyme kinetics. However, after twelve hours of reaction, we observed a decrease in doping levels, which was more significant in the case of 0.5 mol% of Hb concentration. When considering the 0.2 mol% of Hb concentration, there is still a noticeable reduction in doping levels. This is consistent with the observations in the case of hemin, as the Hb slowly degrades, producing free Fe^3+^ ions, which can de-dope the n-PBDF. To investigate the mechanism of n-PBDF formation using hemoproteins, we selected Mb and 2-coumaranone (BF) as our model system. When Mb is mixed with BF at 37 °C with and without surfactants, we see the rise of two prominent peaks at 542nm and 580nm (Figure S8 and S9) corresponding to the ferryl Mb.^30^ Furthermore, these ferryl Mb peaks only occur in the presence of either BF or BFD and oxygen. To confirm the formation of ferryl Mb, we further conducted Raman measurements of Mb in the presence of BF at 37 °C (Figure S10). We see the decrease in the peak intensity at 1560 cm^−1^ corresponding to the Fe^3+^-Mb and the rise of a tiny peak at 780 cm^−1^ corresponding to the ferryl Mb.^31^ Hence, the proposed radical-based mechanism and the catalytic cycle are represented in Figure 3d.

## Whole Blood Catalyzed Polymerization of n-PBDF

After successfully polymerizing n-PBDF using lyophilized hemoproteins, we investigated the potential of naturally occurring Hb in RBCs to catalyze the oxidative polymerization of n-PBDF. Upon comparing the isolated lyophilized bovine Hb powder and freshly isolated RBCs (erythrocytes) from female human blood, it was observed that there was hardly any noticeable difference between the two, with similar conversion and reaction rates in RPMI media (Figure 3a). Using freshly obtained human female whole blood as the catalyst yielded a significantly higher conversion and doping level than using isolated RBCs (Figure 3b & Figure S11), proving the robustness of the catalytic system. After conducting a more thorough investigation into the polymerization of n-PBDF with whole blood, we observed that a minimum concentration of 5 mM BDF is necessary for n-PBDF formation when BDF concentration was varied from 0.1 mM to 50 mM (Figure 3c & Figure S12) while using 12.5 μL of whole blood. Additionally, we noticed a slight decrease in the doping level of the n-PBDF polymer when the quantity of whole blood was increased (Figure S13). This result is consistent with our previous findings when utilizing lyophilized Hb as the catalyst. To evaluate the efficacy of polymerization without the need for constant stirring, we employed a method whereby the solution was placed in an incubator with a rocker at 37 °C. Upon introduction of whole blood into the reaction vessel without stirring, we observed a sluggish reaction, and the vessel turned black after three hours into the reaction. Additionally, we observed that the conversion rate was lower in a closed-cap vessel but improved significantly in the presence of an air balloon (Figure S14). Based on our observations from the reaction without stirring, we deduced the lysis of the RBCs with the BDF monomer. To confirm, we proceeded with a hemolysis assay on the various reaction components (Figure S15a) and with different BDF concentrations (Figure S15b). We found that 5% v/v DMSO and 1% w/w TPGS caused less than 1% of red blood cells to lyse. The BDF monomer resulted in significant hemolysis of approximately 40% at 0.2 mg/mL. This explains the initial slow reaction and formation of n-PBDF without stirring. Because the BDF monomer can lyse the RBCs, we further studied the cytotoxicity of the BDF monomer and n-PBDF (Figure S16) in A549 lung cancer cells. We observed that the n-PBDF polymer is non-toxic to cancer cells, with cell viability remaining close to 100% regardless of the polymer concentration (Figure S16b). However, as the concentration of monomer increased from 5 μg/mL to 0.167 mg/mL, the viability of cells decreased significantly from approximately 75% to around 10%, respectively. This indicates that the monomer is toxic to cancerous cells, as demonstrated by a 72-hour assay. Similarly, on the 6-hour assay, we saw similar results with slightly improved cell viability at higher concentrations of BDF monomer (Figure S16a). Although the BDF monomer is toxic in cancer cells, it may not impede the *in vivo* polymerization process, as once the polymerization reaction begins, the toxicity quickly diminishes.

## *In Vivo* Polymerization of n-PBDF in Zebrafish Embryos

**Figure 4.**
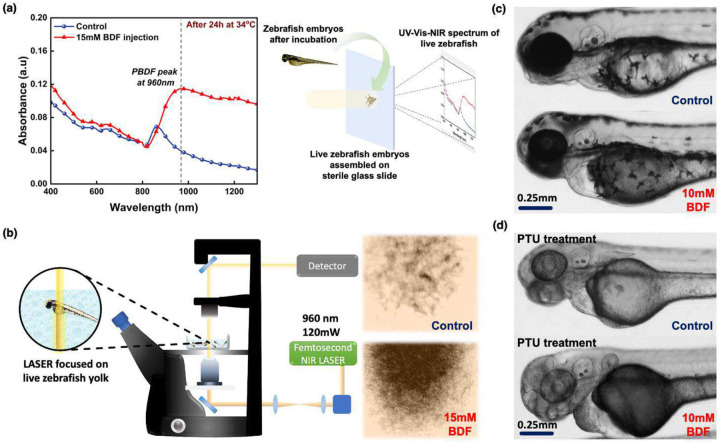
Blood catalyzed polymerization of *n*-PBDF in living Zebrafish embryos at 34°C. **a)** UV-Vis-NIR spectrum of live zebrafish embryos after 24h incubation, **b)** NIR absorption images of live zebrafish embryos after 24h incubation with 960nm LASER focused on yolk, Microscope image of zebrafish embryos with darkened yolk after 24h incubation **c)** without and **d)** with PTU treatment. (The results are representative of at least three unique experiments, each conducted with 20 zebrafish embryos)

Finally, BDF monomer with 1% w/w TPGS in PBS solution was injected in the vasculature of 3-day post-fertilization zebrafish embryos to validate the whole blood catalyzed *in vivo* polymerization of n-PBDF. The fish embryos were injected with different BDF concentrations from 1 mM to 15 mM and kept inside an incubator at 34 °C. Only the 5 mM concentration and above resulted in the darkening of the yolk after 24 hours (Figures 4c, S17, & S19). These results match the minimum BDF concentration required for efficient n-PBDF polymerization using whole blood as the catalyst. To prevent the misconception of pigmentation in the skin of the embryos as a darkened yolk, zebrafish embryos were treated with 1-phenyl 2-thiourea (PTU). PTU treatment removes the pigment from the skin, allowing for better visualization of the darkening of the embryo’s yolk (as shown in Figures 4d, S18 & S20). In the case of a 1 mM BDF concentration injection, we observed a clear yolk similar to that seen in control zebrafish embryos. Significant darkening happened when the concentration was increased to 10 mM. Furthermore, we injected 15 mM BDF concentration into the zebrafish embryo, where the yolk is even darker with respect to the 10 mM BDF concentration injection (Figure S21). To characterize the darkened yolk, we collected UV-Vis-NIR measurements on all zebrafish embryos with different concentrations of BDF injections along with the control fish (only PBS injection) (Figure 4a). When a 15 mM BDF concentration is injected into the zebrafish embryo, a noticeable peak at 960 nm is observed after 24 hours of incubation, indicating the presence of an un-doped PBDF, and providing some indication of the *in-vivo* polymer formation from the BDF monomer. Only in the case of 15 mM BDF injection, the PBDF peak is prominent, while in the case of 10 mM injection, the PBDF peak, although present, is overshadowed by the broadening of the peak while overlapping with the peak at 840 nm (Figure S23). To further verify the formation of PBDF, we used a 960nm NIR femtosecond LASER focusing on the yolk of the incubated zebrafish embryos. The absorption images show higher absorption in the case of 15 mM BDF-injected embryos than the control zebrafish embryos (Figure 4b and S24). It is noted that BDF injection and *in vivo* polymerization of PBDF inside zebrafish embryos showed almost no toxicity (Figure S22), with at least 80% of the embryos alive after 24h incubation and showing movement similar to the control embryos. Upon close examination, the beating heart of the embryos can be confirmed under a microscope, for both without and with PTU-treated zebrafish embryos.

## Non-Genetic Neural Stimulation Using *In Cellulo* Synthesized n-PBDF

**Figure 5.**
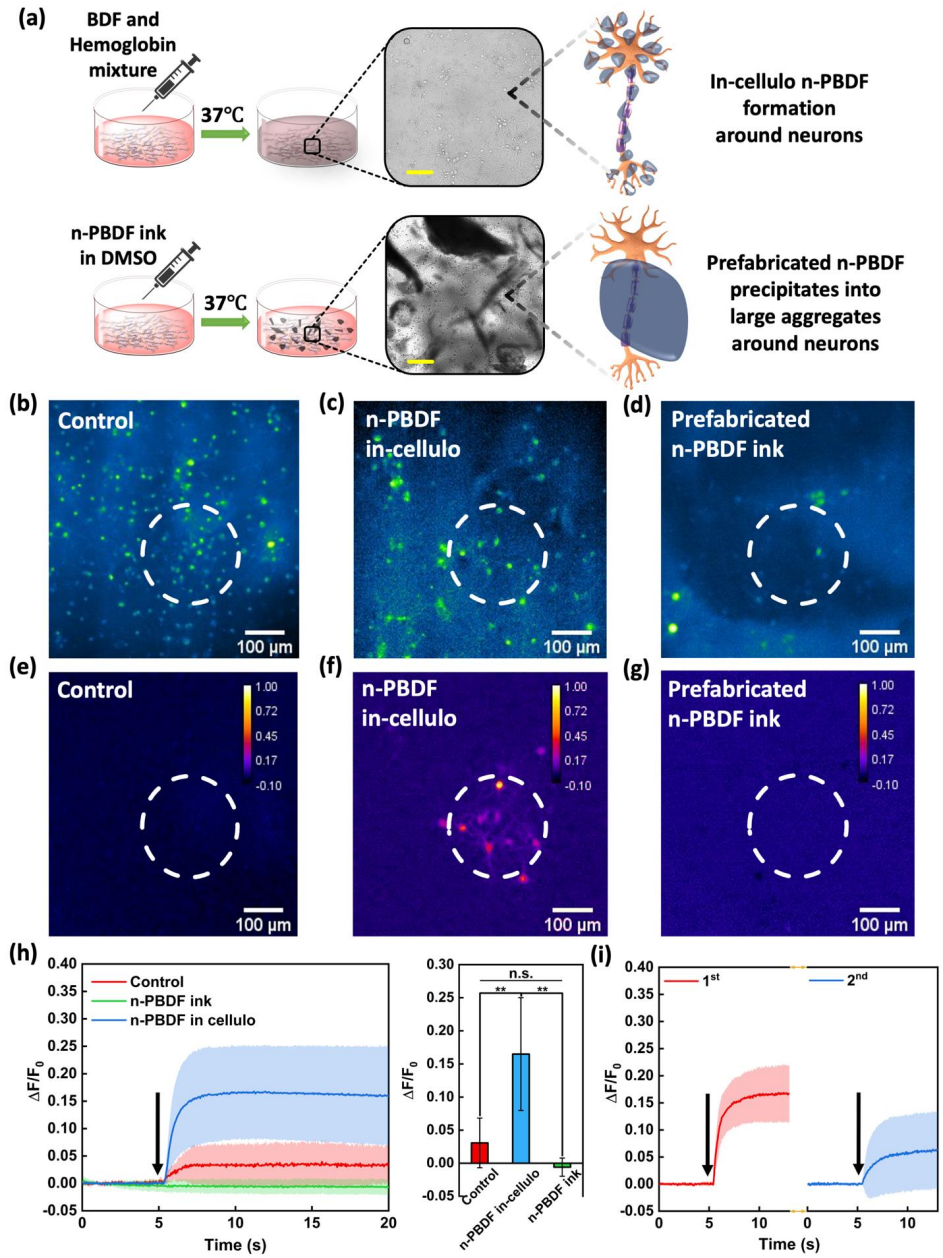
Non-genetic neural stimulation in cultured primary neurons using *in cellulo* formed n-PBDF. **a)** Comparison of *in cellulo* formed n-PBDF using Hb with prefabricated n-PBDF ink on cultured neurons. **b-d)** Representative fluorescence images of Oregon Green labeled neurons upon a nanosecond 1030 nm pulse laser with **b)** no polymer applied, as control, **c)** *in cellulo* formed n-PBDF, and **d)** prefabricated n-PBDF ink. White dashed circles indicate the illumination area of the laser. **e-g)** Max ΔF/F_0_ images of neurons corresponding to Figure 5b–d. **h)** Left panel: representative average calcium trace of neurons in control (n=9), n-PBDF *in cellulo* (n=26), and n-PBDF ink (n=18) groups, respectively. Shaded areas represent one standard deviation. Black arrow: laser onset. Right panel: Average of ΔF/F_0_. Error bars represent standard deviation (n > 9, **p < 0.01, n.s. p = 0.42, one-way ANOVA and Tukey’s mean comparison test). **i)** Average calcium trace for two sequential stimulations of neurons in *in cellulo* n-PBDF within the illumination area (n=13). Shaded areas represent one standard deviation. Black arrows: laser onset. The time interval between two sequential stimulations was 1 min. Laser condition used for n-PBDF *in-cellulo* and n-PBDF ink groups: 30 μJ pulse energy, 3 ms duration, 1.7 kHz repetition rate. Laser condition for the control group: 30 μJ pulse energy, 1 s duration, 1.7 kHz repetition rate.

To unlock the full potential of our method of *in vivo* n-PBDF synthesis using endogenous catalysts, we explored their possible biomedical applications, specifically in neural stimulation. A recent report showed that polymer nanoparticles with modified negatively charged surfaces can bind to neuronal membranes efficiently and can be used as a photoacoustic nanotransducer to modulate the activities of neurons.^32^ Taking advantage of the inherent property of n-PBDF having high absorption in the NIR range and being negatively charged while synthesized *in situ*, we studied its potential application of *in cellulo* formed n-PBDF to enable non-genetic neural stimulation in cultured primary neurons. We first investigated whether *in situ* formed n-PBDF can bind to neuron membranes. 1 mL 0.1mg/mL BDF and 10μM Hb were added to embryonic cortical neurons (10–14 days) in vitro (DIV) from Sprague-Dawley rats (Figure 5a). We used label-free transient absorption (TA) microscopy to visualize the binding of *in situ* formed n-PBDF to neurons, as n-PBDF shows strong intrinsic TA signals (Figure S25). After 16 hours of incubation, the *in situ* formed n-PBDF was primarily found to be bound to the neuronal membrane (Figure S25). In addition, the transmission microscopy images of neuron culture with *in situ* formed n-PBDF and prefabricated n-PBDF ink in DMSO showed different features (Figure 5a). The *in situ* formed n-PBDF dispersed uniformly on neurons. However, the prefabricated n-PBDF ink formed large aggregates in neuron-cultured media and didn’t interact well with the neurons, as shown in Figure 5a.

We performed calcium imaging on Sprague-Dawley rat primary cortical neurons labeled with Oregon Green 488 BAPTA-1 to evaluate *in cellulo* formed n-PBDF potential for neural stimulation. A 1030 nm nanosecond laser with a pulse width of 3 ns, repletion rate of 1.7 kHz, and pulse energy of 30 μJ was delivered to neurons. The laser duration was set to 3 ms. Activation of the neurons was characterized by the fluorescence intensity changes in fluorescence (ΔF/F_0_) of neurons during stimulation. As shown in Figures 5c, 5f, 5h, and real-time video (Video S1), an increase in fluorescence intensity in neurons cocultured with *in cellulo* formed n-PBDF was observed immediately after laser onset, indicating successful stimulation of neurons. The laser only control group showed less fluorescence intensity increase after applying the same NIR laser with an even higher duration of 1s, as shown in Figures 5b, 5e, and 5h. The neurons cultured with prefabricated n-PBDF ink showed almost no response, as shown in Figures 5d, 5g, and 5h, and real-time video (Video S2), which could be attributed to the weak interaction of n-PBDF with the neurons due to the formation of large aggregates. Furthermore, it is worth noting that no activations were observed outside the illuminated area of the laser (Figure 5e–g), confirming stimulation as triggered by light in the presence of only *in cellulo* formed n-PBDF. Calcium traces further confirmed that the average ΔF/F_0_ in *in cellulo* formed n-PBDF group was 17%. Much less ΔF/F_0_ change was observed in both the control and prefabricated n-PBDF ink groups. Our result showed that activation of neurons was enabled by *in cellulo* formed n-PBDF through a light-triggered neural stimulation.

A repeated PA stimulation by irradiating neurons with n-PBDF *in cellulo* was also performed to validate the reliability of photoacoustic stimulation. Two sequential 1030 nm pulsed laser was delivered. Each had a duration of 3 ms. A 1-minute interval was applied between two stimulations to allow recovery of fluorescence signals of neurons to the baseline. Figure 5i shows successful activation was observed on the same group of neurons after each stimulation. This result indicates stimulation induced by *in cellulo* n-PBDF is repeatable and reliable. n-PBDF has a very high absorption coefficient in the NIR region, and NIR light has been shown to penetrate tissue and possibly the human skull.^33^ Hence, n-PBDF formed *in cellulo* presents an exciting opportunity for neural modulation, opening up the potential for non-surgical brain stimulation through light excitation.

## Conclusion

The conductive polymer n-PBDF is synthesized in a biological environment with natural enzymes (hemoproteins), and inside live zebrafish embryos. The *in cellulo* formed-PBDF exhibits enhanced bio-interfaces with primary cultured neurons for effective neural activation. Coupled with its simplicity and superior biocompatibility, this innovative approach of polymerization inside living organisms opens up promising prospects for its application in future biomedical innovations.

## Methods

### Materials:

All reagents for the synthesis and analysis were purchased from Sigma Aldrich, Alfa Aesar, Acros Organics, Oakwood Chemical, and TCI Chemicals and used without further purification unless otherwise mentioned. Solvents were purchased from Fisher Scientific and used without any further purification. BDF precursor acid (2,5-dihydroxy-1,4-benzenediacetic acid) was purchased from Santa Cruz Biotechnology and was recrystallized twice in water under nitrogen.

#### UV-Vis-NIR Absorption Spectroscopy:

UV–vis–NIR absorption spectra were measured with an Agilent Technologies (Cary 5000/6000i) Cary Win UV–vis–NIR spectrophotometer. Solution UV–vis–NIR spectra were recorded in a quartz cuvette with a 1 cm path length. UV-vis-NIR absorption spectra for RBCs/Blood samples were measured with a ThermoScientific Genesys 30 Visible spectrophotometer. RBCs/Blood solution UV-vis-NIR spectra were recorded in a polystyrene disposable cuvette with a 1 cm path length.

#### Conductivity and Sheet Resistance Measurements:

Sheet resistance was measured through the top-contact four-probe measurement in Filmetrics R50-200-4PP Resistance Mapper. To obtain uniform drop-casted thin-film samples, the polymer solutions were sonicated in an ultrasonic bath for 5 min and then stirred for 2 hours at room temperature. Before drop casting, all polymer samples were treated by a vortex mixer for 5 mins. Polymer inks (200 μL) were deposited on the clean Si/SiO_2_ substrates and were then placed in a vacuum oven at room temperature for at least 12 hours for the complete removal of the residual solvent. After that, the thin films were annealed at 80°C for 15 mins under a vacuum to remove any residual surfactants from the thin films and then kept under nitrogen flow until the substrates cooled down to room temperature. The conductivity of the films was then calculated by the equation below:

$$\sigma =\frac{1}{R_{s}t}$$

Where *R*_*s*_ is the measured sheet resistance, *t* is the film thickness (measured by a profilometer). A minimum of 3 replicates were measured for consistent results.

#### Hemolysis Assay:

Hemolysis assays were based on a previously described method.^34^ Human Whole Blood was purchased from BioIVT and used before its expiration date. 100 μL of blood was aliquoted into a 1.5 mL Eppendorf tube, and 500 μL of sterile 0.9% NaCl was added. Tubes were gently inverted to mix and then centrifuged at 500Xg for 7 minutes. The supernatant was carefully removed, and the pellet was washed 2X with 500 μL of 0.9% NaCl. The pellet was then resuspended in 800 μL of Red Blood Cell (RBC) buffer (10 mM Na_2_HPO_4_, 150 mM NaCl, 1 mM MgCl_2_, pH 7.4). To evaluate the hemolytic activity of compounds, they were added at desired concentrations (3% DMSO final) and transferred to a 96 U-well plate well. Negative control wells contained 4 μL of DMSO, and positive controls contained 4 μL of 30% Triton X-100. In each well was added 76 μL of RBC buffer and 40 μL of the resuspended red blood cells. This was incubated for 1h at 37 °C. The plate was then spun at 500Xg for 5 min, and the supernatants from each sample (75 μL) were transferred to a flat-well 96-well plate. The absorbance of these supernatants at 540 nm was then measured using a SpectraMax iD3 plate reader. Percent hemolysis was calculated relative to the average absorbance values for the positive and negative controls. A minimum of three biological replicates was performed.

#### SRB Cytotoxicity:

Cytotoxicity assays were based on a previously described method.^35^ A549 non-small cell lung cancer cells (ATCC CCL-185) were obtained directly from ATCC and used within 20 passages. A549 cells were maintained in RPMI 1640 medium supplemented with 10% fetal bovine serum, 100 U mL^−1^ penicillin, and 100 ug mL^−1^ streptomycin. For cytotoxicity testing, cells were seeded at 20,000 cells per well and allowed to adhere overnight. Cells were then treated with compound at desired concentrations (1% DMSO final) or vehicle control for 72 hours at 37°C. Viability was assessed utilizing Sulfurhodamide B after a 1-hour incubation period. Absorbance was measured using a SpectraMax iD3 plate reader (At 510 nm). Percent death was calculated by subtracting the background from all wells and setting 0% death to controls.

#### SRB Pulse Cytotoxicity:

A549 non-small cell lung cancer cells (ATCC CCL-185) were obtained directly from ATCC and used within 20 passages. A549 cells were maintained in RPMI 1640 medium supplemented with 10% fetal bovine serum, 100 U mL^−1^ penicillin, and 100 ug mL^−1^ streptomycin. For pulse cytotoxicity testing, cells were seeded at 20,000 cells per well and allowed to adhere overnight. Cells were then treated with the compound at desired concentration (1% DMSO final) or vehicle control. After a 6-hour incubation period at 37°C, the cells were washed with warmed phosphate-buffered saline (PBS) three times and incubated for 24 hours in RPMI media with 10% fetal bovine serum, 100 U mL^−1^ penicillin, and 100 ug mL^−1^ streptomycin. Cell viability was assessed utilizing Sulfurhodamide B after a 1-hour incubation period. Absorbance was measured using a SpectraMax iD3 plate reader (At 510 nm). Percent death was calculated by subtracting the background from all wells and setting 0% death to controls.

#### Zebrafish blood injection assay (PTU Treated):

The zebrafish husbandry and experiment were conducted in accordance with internationally accepted standards. The Animal Care and Use Protocol was approved by The Purdue Animal Care and Use Committee (PACUC), adhering to the Guidelines for using Zebrafish in the NIH Intramural Research Program (protocol number: 1401001018). The AB wild-type strain was used in this study. The zebrafish blood injection in this study is modified from the zebrafish vasculature infection assay as described previously.^36^ The larvae were treated by 200uM PTU (1-phenyl 2-thiourea) in E3 medium starting at 24hrs post-fertilization for inhibiting pigmentation on the fish body. Briefly, 20 of 3 days post fertilization larvae in each group were injected with 1nL of desired concentration of BDF monomer solution into the ventral vein while the sterile PBS was injected as control. Then the fish larvae were incubated at 34°C for 24hr before imaging. After 24 hours post-injection, the zebrafish were imaged by a Zeiss ZV16 microscope with a bright field and pooled by the group for downstream analysis. Representative experiments of three independent repeats were shown.

#### Zebrafish blood injection assay:

The zebrafish husbandry and experiment were conducted in accordance with internationally accepted standards. The Animal Care and Use Protocol was approved by The Purdue Animal Care and Use Committee (PACUC), adhering to the Guidelines for using Zebrafish in the NIH Intramural Research Program (protocol number: 1401001018). The AB wild-type strain was used in this study. The zebrafish blood injection in this study is modified from the zebrafish vasculature infection assay as described earlier. Briefly, 20 of 3 days post fertilization larvae in each group were injected with 1nL of the desired concentration of BDF monomer solution into the ventral vein while the sterile PBS was injected as control. Then, the fish larvae were incubated at 34°C for 24 hours before imaging. After 24 hours post-injection, the zebrafish were imaged by a Zeiss ZV16 microscope with a bright field and pooled by the group for downstream analysis. Representative experiments of three independent repeats were shown.

### Animals:

All experimental procedures complied with all relevant guidelines and ethical regulations for animal testing and research established and approved by the Institutional Animal Care and Use Committee (IACUC) of Boston University (PROTO201800535). Primary cortical neurons were isolated from embryonic day 15 – 18 (E15-E18) Sprague–Dawley rat embryos of either sex (Charles River Laboratories, MA, USA).

### Embryonic Neuron Culture:

The glass-bottomed culture dishes used in the embryonic neuron cell cultures were immersed in 0.01% poly-D-lysine (Sigma-Aldrich) overnight at 37 °C and washed in PBS before culture initiation. Primary cortical neurons were obtained from Sprague-Dawley rats. Cortices were dissected from E15-E18 rats of either sex and digested in TrypLE Express (Thermo Fisher) for 15 min at 37°C and triturated every 5 min. Dissociated cells were washed with and triturated in 10% heat-inactivated fetal bovine serum (FBS) (Atlanta Biologicals), 2 mM glutamine-Dulbecco’s modified Eagle’s medium (DMEM) (Thermo Fisher) and cultured in cell culture dishes (100mm diameter) for 30 min at 37°C to eliminate glial cells and fibroblasts. The supernatant containing neurons was collected and seeded on a poly-D-lysine-coated cover glass and incubated in a humidified atmosphere containing 5% CO_2_ at 37°C with 10% FBS + 2 mM glutamine-DMEM. After 16h, the medium was replaced with a growth medium containing 2% B27, 1% N2, and 2 mM glutamine (Thermo Fisher). Half of the medium was replaced with fresh growth medium every 3 – 4 days. Neurons cultured *in vitro* for days 10 – 14 were used for the following experiments.

### Transient Absorption (TA) Microscopy:

TA images were obtained as previously described.^37^ For each TA image, the Z position of the focus was adjusted near the equatorial plane of the neurons so that the soma and neurites were both visualized. The powers of the pump (1,045nm) and probe (845nm) were maintained at 20mW. Both the pump and probe beams were linearly polarized. No cell or tissue damage was observed. Images were acquired at a pixel dwell time of 2μs.

### In Vitro Neurostimulation:

n-PBDF ink in DMSO or BDF/Hb solution in neural media was added into the culture medium of neurons to reach a final concentration of 0.3 mg/mL. An incubation time of 6 h and 24 h was tested. A Q-switched 1030nm nanosecond LASER (Bright Solution) with 3 ns pulse width and 1.7 kHz repetition rate was used. The LASER was delivered using an optical fiber (Thorlabs) with a diameter of 200μm and 0.22 NA. Before the stimulation experiment, the cultured neurons were labeled with Oregon Green 488 BAPTA-1. The fiber was placed approximately 100μm above the neurons during neurostimulation experiments. Calcium fluorescence imaging was performed on a lab-built wide-field fluorescence microscope. The microscope was based on an Olympus IX71 microscope frame with a 10x air objective (UPLSAPO20x, 0.75 NA; Olympus) illuminated by a 470nm LED (M470L2; Thorlabs), and a dichroic mirror (DMLP505R; Thorlabs). Image sequences were acquired with a scientific CMOS camera (Zyla 5.5; Andor) at 20 frames per second. The fluorescence intensity analysis and exponential curve fitting were performed using ImageJ (Fiji).

## Supplementary Material

1
