# Supplementary material for: Blood-Catalyzed Polymerization Creates Conductive Polymer in Live Zebrafish
Source: Res Sq. 2024 Jun 25:rs.3.rs-3602290. Preprint. [Version 1] doi: 10.21203/rs.3.rs-3602290/v1 (PMC11230466; doi:10.21203/rs.3.rs-3602290/v1)
Supplement: 1 [file NIHPPRS3602290V1-supplement-1.pdf]

Supporting Information for

Blood-Catalyzed Polymerization Creates Conductive Polymer in Living Fish

Sanket Samal[1], *Samantha Nelson*[2], *Zhiyi Du*[3], *Decheng Wang*[4], *Tianqi Wang*[4], *Chen Yang*[3], *Qing Deng*[4,5,6], *Elizabeth I. Parkinson*[1,2], *Jianguo Mei*[1*]

[1]Department of Chemistry, Purdue University, West Lafayette, IN, USA.
[2]Department of Medicinal Chemistry and Molecular Pharmacology, Purdue University, West Lafayette, IN, USA.
[3]Department of Chemistry, Boston University, Boston, MA, USA.
[4]Department of Biological Sciences, Purdue University, West Lafayette, IN, USA.
[5]Purdue Institute for Inflammation, Immunology & Infectious Diseases, Purdue University, West Lafayette, IN, USA.
[6]Purdue University Center for Cancer Research, Purdue University, West Lafayette, IN, USA.

Jianguo Mei
Email: jgmei@purdue.edu

This PDF file includes:

Synthetic scheme of BDF monomer and n-PBDF polymer
Supplementary Figures S1 to S26

Experimental Section

Synthesis

***Synthesis of BDF monomer*:** BDF was synthesized via a previously reported method with slight modification.[1]

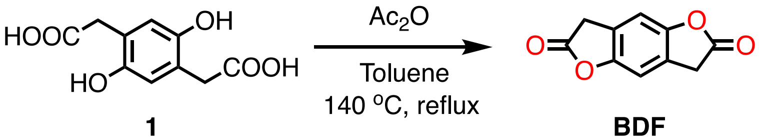

1 **BDF**

To a suspension of compound **1** (1.0 g, 4.4 mmol) in anhydrous toluene (15 mL), Ac_2O (15 mL) was added. The mixture was stirred at 140 °C for 5 h, and then the reaction was cooled to room temperature slowly overnight. The recrystallized product was filtered under a vacuum and washed with cold toluene at least three times. The product was kept under a high vacuum at room temperature to give **BDF** as a white crystal (0.65 g, 78 %). ^{1}H NMR (DMSO-D_6, 400 MHz, ppm): δ 7.24 (s, 2H), 3.95 (s, 4H).

***Synthesis of n-PBDF polymer with copper acetate*:** The BDF monomer was dissolved in a stock solution of 5% DMSO in PBS buffer with 1% w/v TPGS-750-M surfactant for 1 hour. After 1 hour, $Cu(OAc)_2$ was added to the solution mixture. The mixture was heated to 37°C and stirred for 6 hours in the air. After the solution was cooled to RT, the mixture was dialyzed against 20% DMSO in DI water using a dialysis bag with a cut-off molecular weight of 10 kDa (ThermoScientific SnakeSkin Dialysis Tubing, USA) to remove low molecular weight fractions, copper catalyst, and additives.

***Synthesis of n-PBDF polymer with Hemin*:** The BDF monomer was dissolved in a stock solution of 5% DMSO in PBS buffer with 1% w/v TPGS-750-M surfactant for 1 hour (25mM BDF concentration). After 1 hour, Hemin was added to the solution mixture. The mixture was heated to 37°C and stirred for 6 hours in the air. After the solution was cooled to RT, the mixture was dialyzed against 20% DMSO in DI water using a dialysis bag with a cut-off molecular weight of 10 kDa (ThermoScientific SnakeSkin Dialysis Tubing, USA) to remove low molecular weight fractions, hemin, and additives.

***Synthesis of n-PBDF polymer with Hemoproteins*:** The BDF monomer was dissolved in a stock solution of 5% DMSO in PBS buffer (or RPMI 1640 media with 10% FBS and 1% Penicillin-streptomycin) with 1% w/v TPGS-750-M surfactant for 1 hour. After that, Hemoprotein was added to the solution mixture. The mixture was heated to 37°C and stirred for 6 hours in the air. After the solution was cooled to RT, the

mixture was dialyzed against 20% DMSO in DI water using a dialysis bag with a cut-off molecular weight of 10 kDa (ThermoScientific SnakeSkin Dialysis Tubing, USA) to remove low molecular weight fractions, hemoprotein, and additives.

Synthesis of n-PBDF polymer with RBCs/Whole Blood**:** The BDF monomer was dissolved in a stock solution of 5% DMSO in PBS buffer (or RBC buffer or RPMI 1640 media with 10% FBS and 1% Penicillin-streptomycin) with 1% w/v TPGS-750-M surfactant for 1 hour. After that, RBCs (suspended in RBC buffer)/Whole Blood were added to the solution mixture. The mixture was heated to 37°C and stirred for 6 hours in the air. After the solution was cooled to RT, characterization was done without any further purification.

Synthesis of n-PBDF polymer using Whole Blood without stirring**:** The BDF monomer was dissolved in a stock solution of 5% DMSO in RPMI 1640 media with 10% FBS,1% Penicillin-streptomycin, and 1% w/v TPGS-750-M surfactant for 1 hour. After that, whole blood was added to the solution mixture. The mixture was kept inside an incubator at 37°C with slow rocking for 8 hours. After the solution was cooled to RT, characterization was done without any further purification.

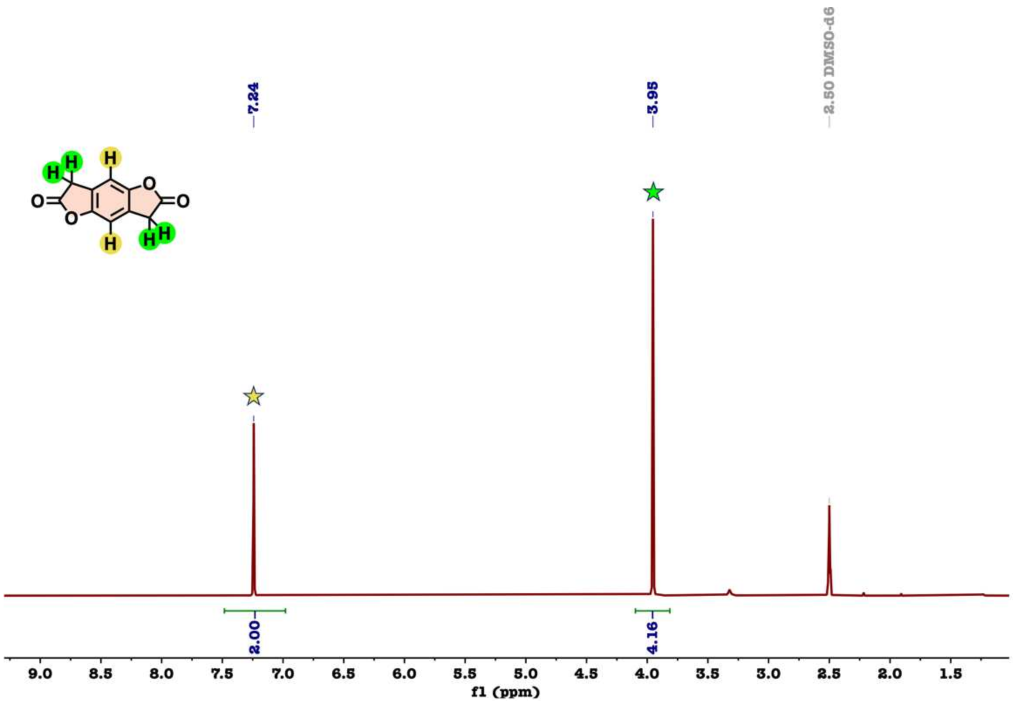

Figure S1. ^{1}H NMR of **BDF** monomer in DMSO-D_6.

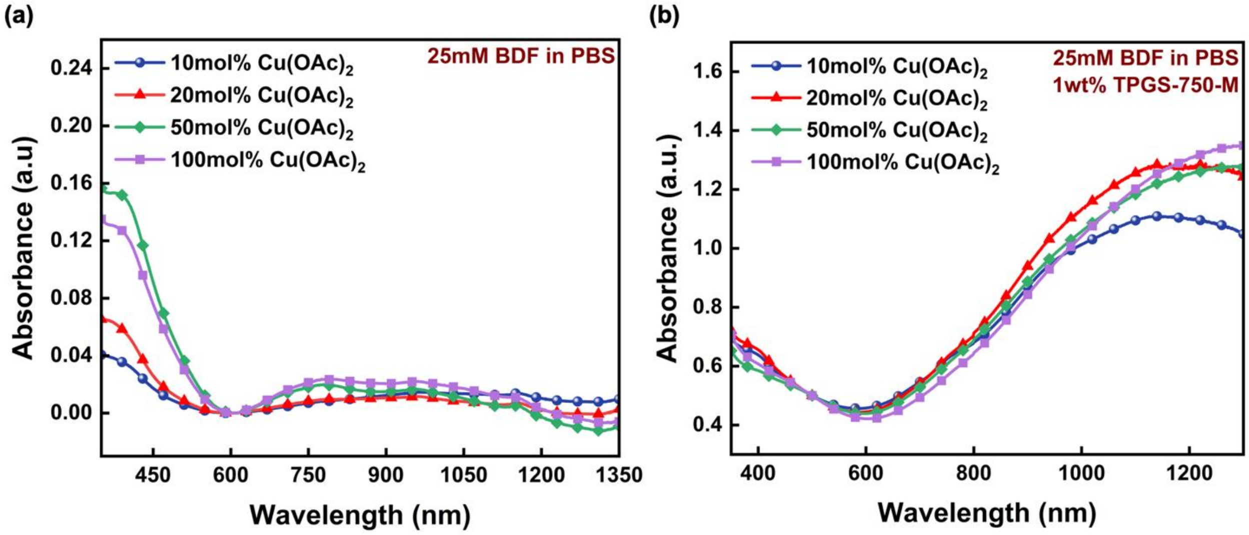

Figure S2. UV-Vis-NIR spectrum of n-PBDF polymer using copper acetate **a)** without surfactant and **b)** with 1wt% TPGS-750-M surfactant.

Using 1wt% TPGS-750-M surfactant, we can observe the formation of highly doped n-PBDF polymer, characterized by a strong polaron peak in the NIR region around ~1150nm, as seen in our previous study.[2] The intensity of the polaron peak increases as the amount of copper acetate increases.

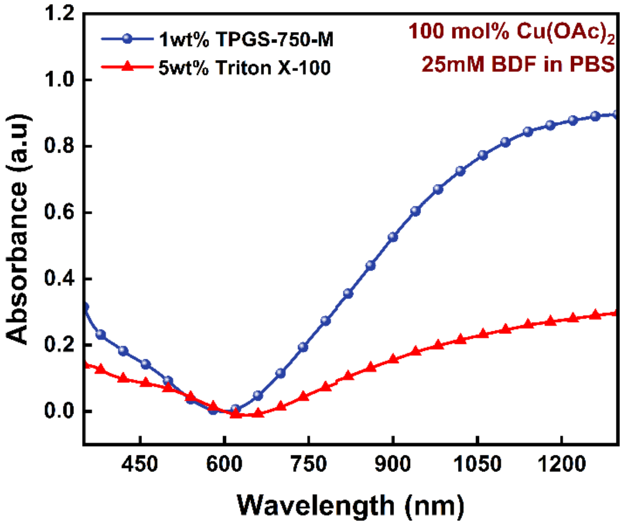

Figure S3. UV-Vis-NIR spectrum of n-PBDF polymer with different surfactants using copper acetate as catalyst.

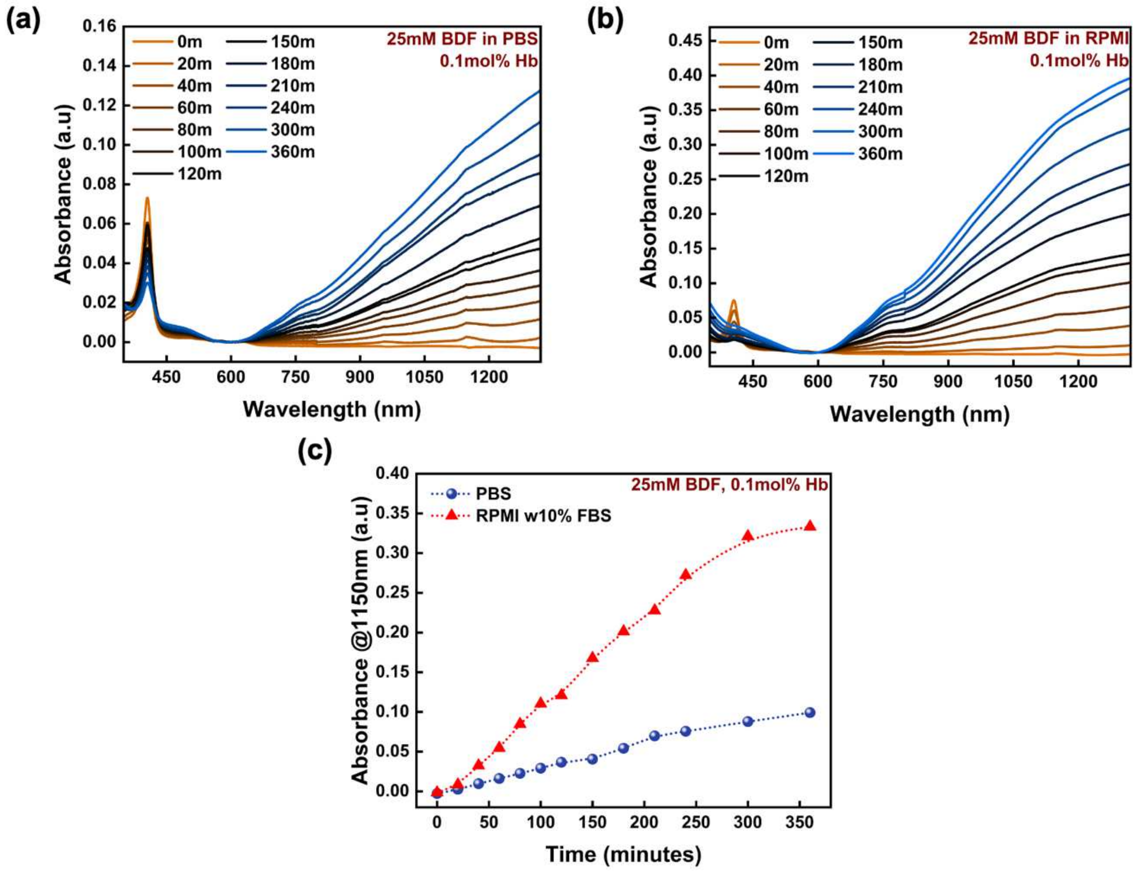

Figure S4. UV-Vis-NIR spectrum of n-PBDF polymer formation using 0.1mol% Hb as catalyst **a)** In PBS Buffer, **b)** In RPMI media, and **c)** comparison of n-PBDF polaron absorption with time in different solvents.

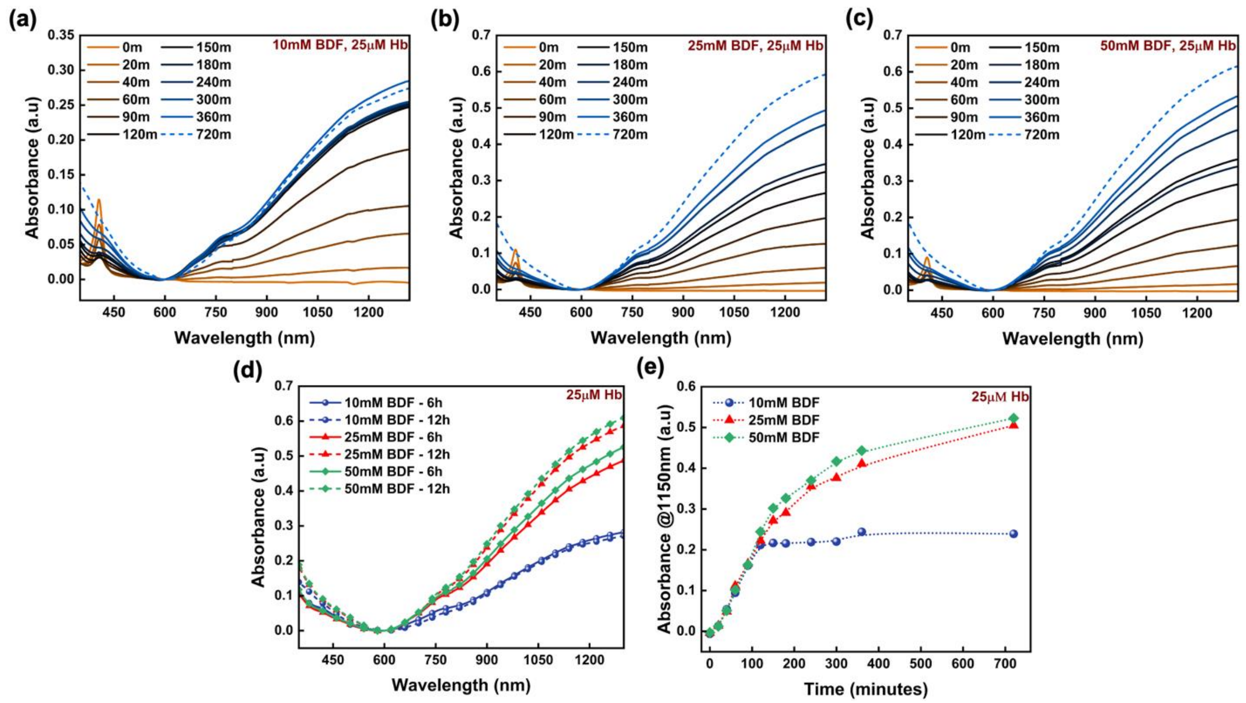

Figure S5. UV-Vis-NIR spectrum of n-PBDF polymer formation using 25μM Hb as a catalyst in RPMI **a)** 10mM BDF, **b)** 25mM BDF, **c)** 50mM BDF, **d)** comparison of n-PBDF polaron absorption at 6h and 12h, and **e)** n-PBDF polaron absorption with time at varying BDF concentrations.

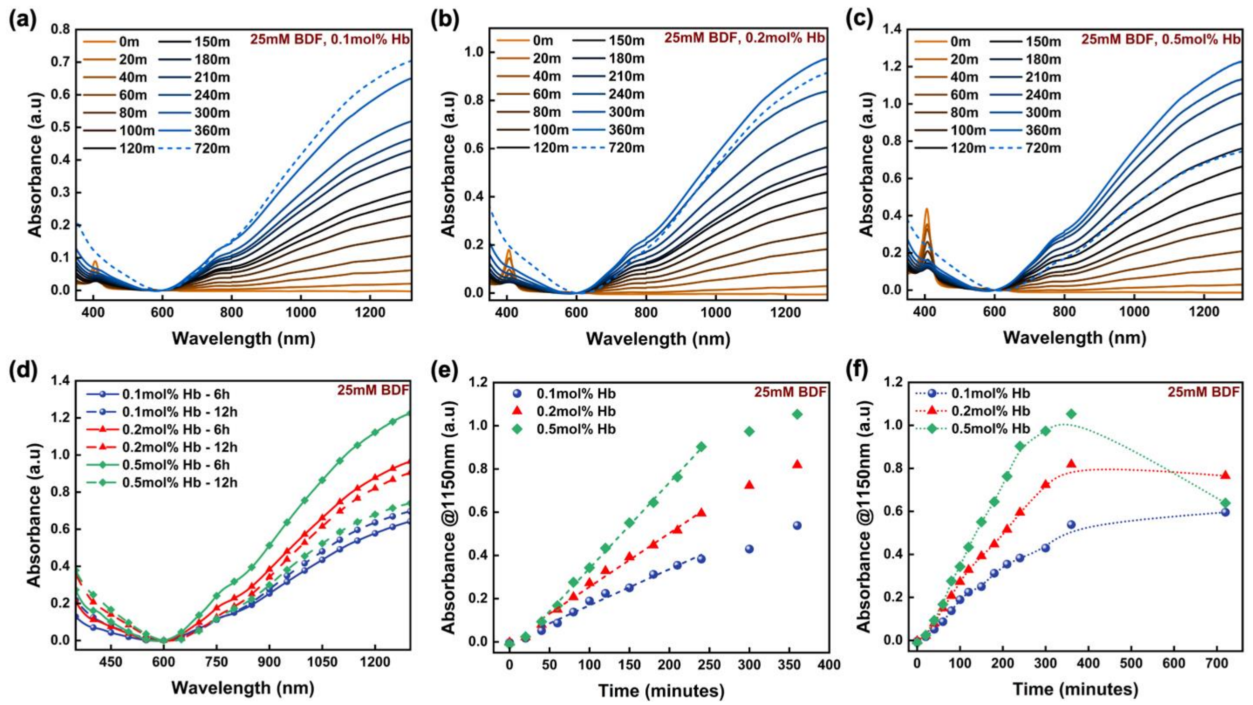

Figure S6. UV-Vis-NIR spectrum of n-PBDF polymer formation using Hb as a catalyst in RPMI with constant BDF concentration of 25mM **a)** 0.1mol% Hb, **b)** 0.2mol% Hb, **c)** 0.5mol% Hb, **d)** comparison of n-PBDF polaron absorption at 6h and 12h, and n-PBDF polaron absorption with time at varying Hb concentrations **e)** In initial 4 hours **f)** In 12 hours.

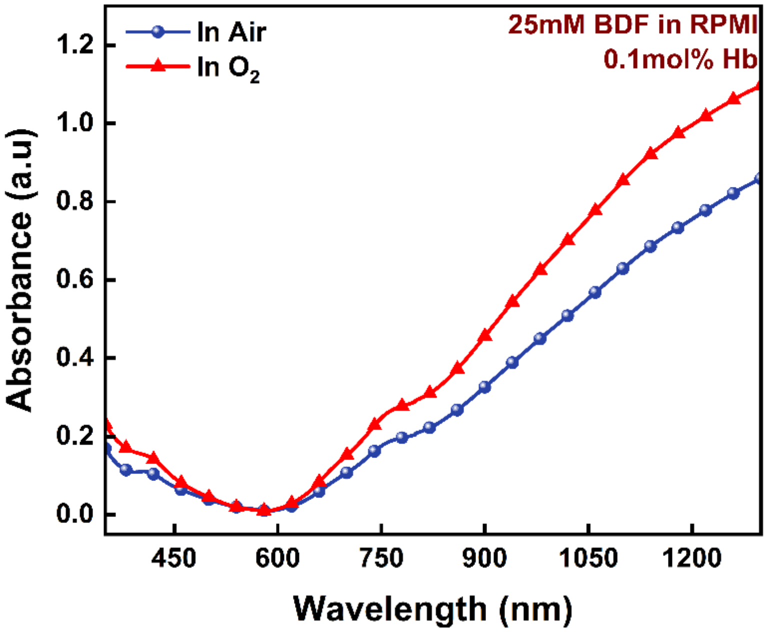

Figure S7. UV-Vis-NIR spectrum of n-PBDF in air with comparison to in pure oxygen atmosphere.

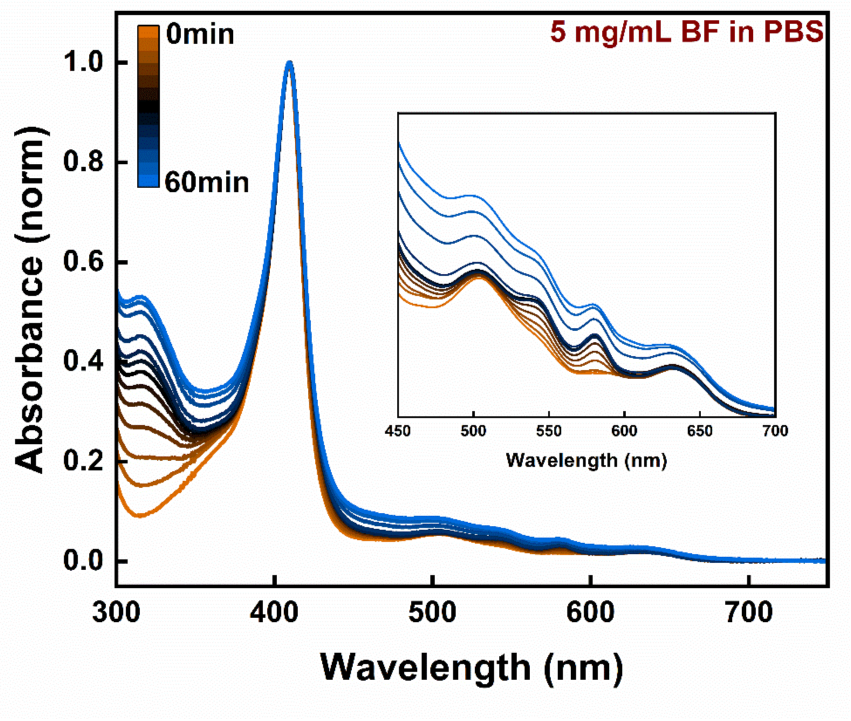

Figure S8. UV-Vis-NIR spectrum of Myoglobin (Mb) along with 2-Coumaranone (BF) in PBS and TPGS surfactant at different intervals at 37 ºC. Inset: zoomed portion from 450nm – 700nm.

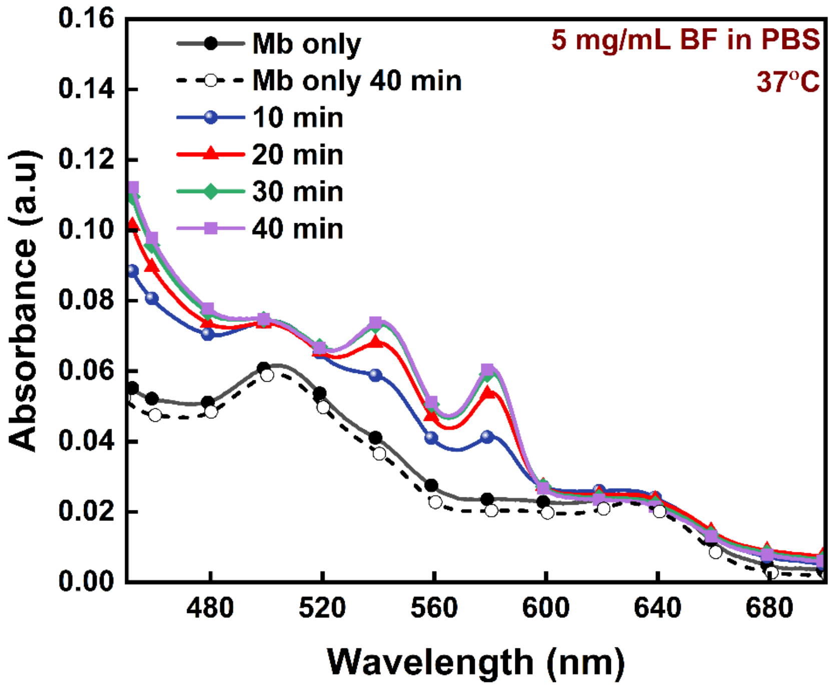

Figure S9. UV-Vis-NIR spectrum of Myoglobin (Mb) along with 2-Coumaranone (BF) in PBS without any surfactant at different intervals at 37 ºC.

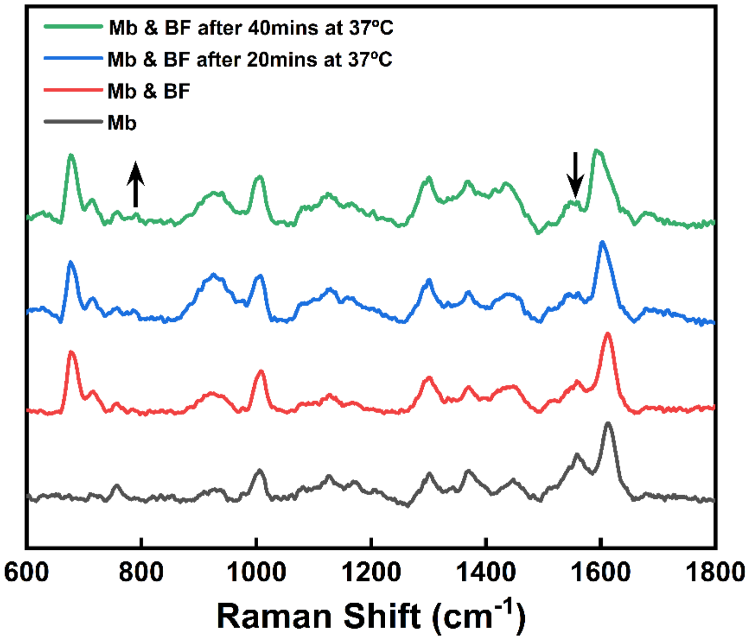

Figure S10. Raman spectrum of Myoglobin (Mb) and 2-Coumaranonr (BF) in PBS with TPGS at different intervals, excitation with 532nm LASER.

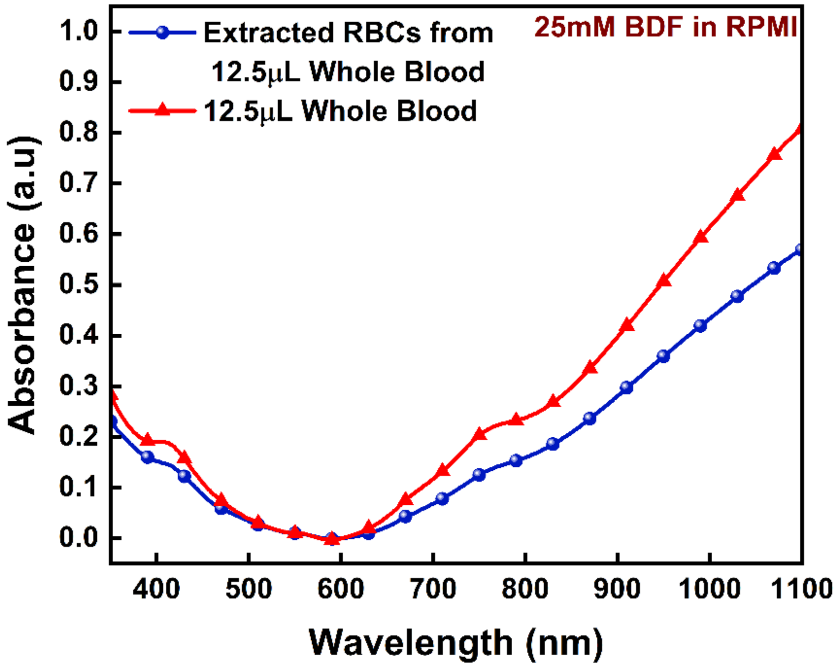

Figure S11. UV-Vis-NIR spectrum of n-PBDF using RBCs compared to whole blood as a catalyst.

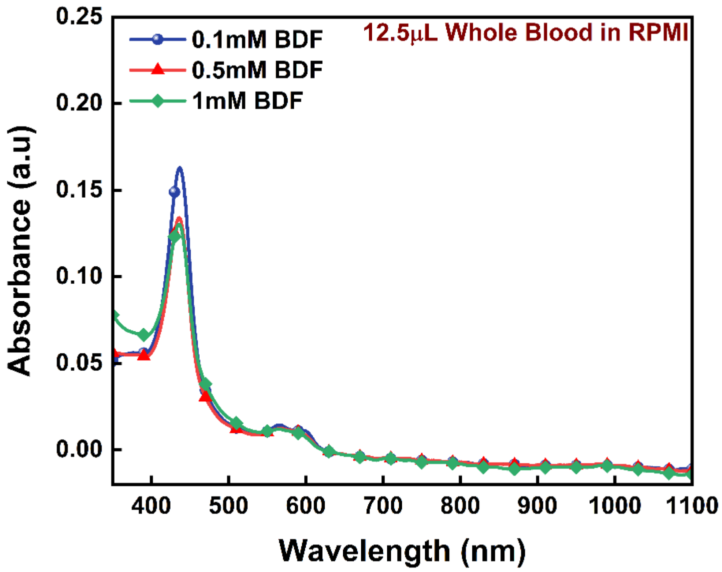

Figure S12. UV-Vis-NIR spectrum of n-PBDF using whole blood as a catalyst at very low BDF concentration.

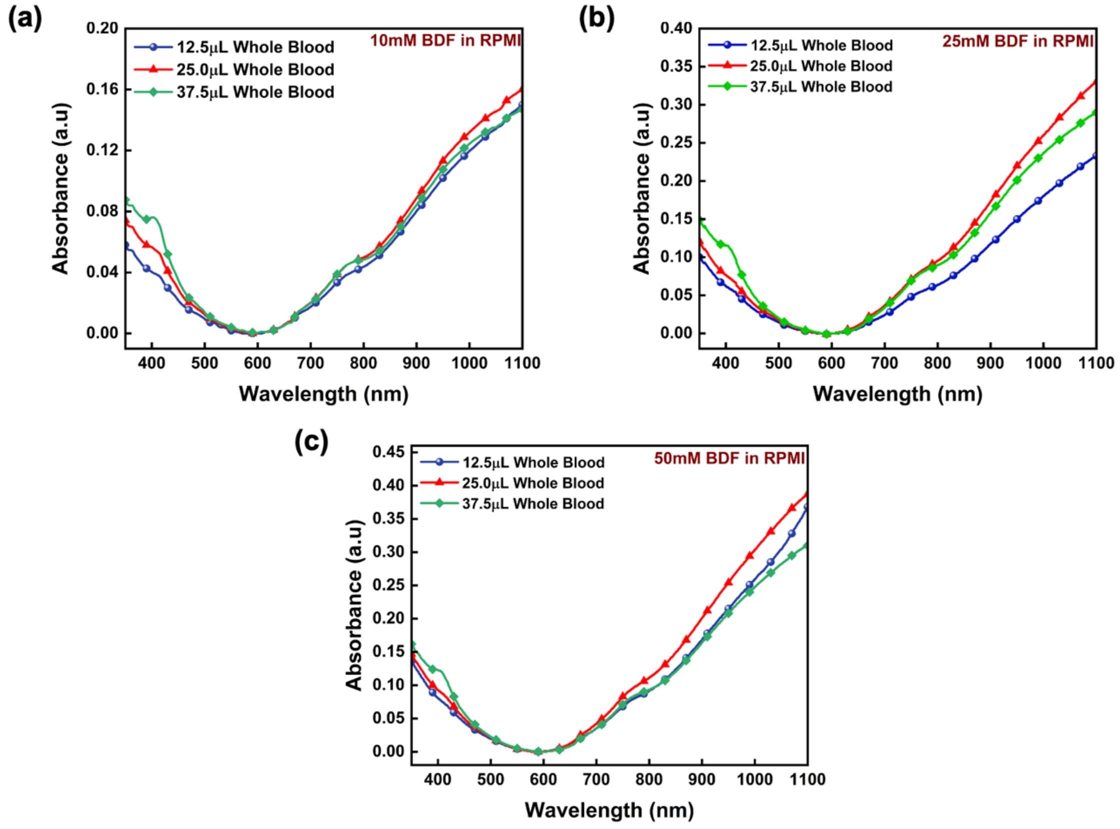

Figure S13. UV-Vis-NIR spectrum of n-PBDF at varying whole blood concentrations with **a)** 10mM BDF, **b)** 25mM BDF, and **c)** 50mM BDF concentrations.

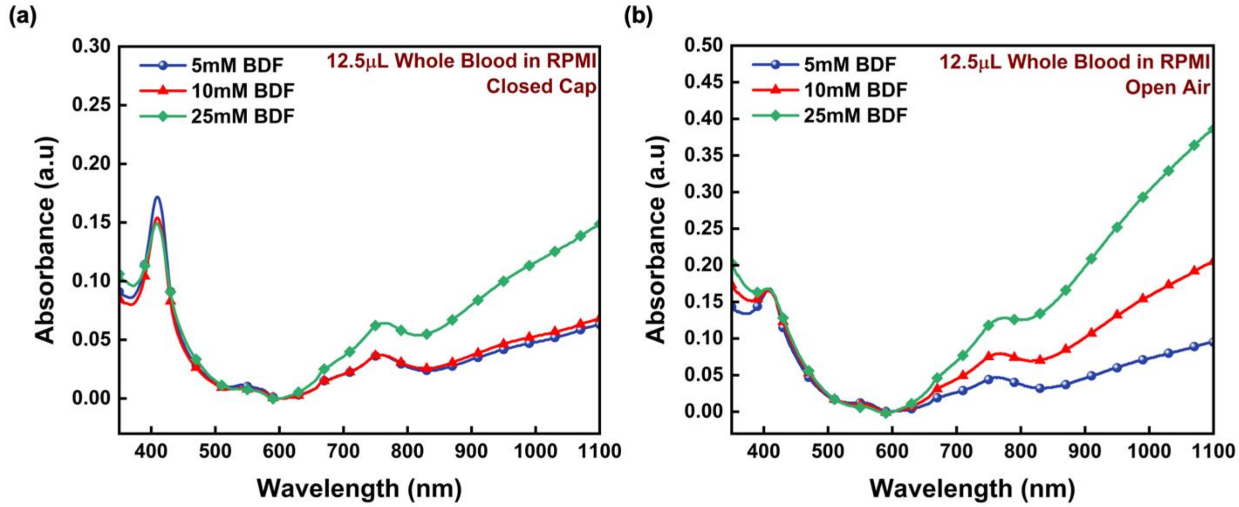

Figure S14. UV-Vis-NIR spectrum of n-PBDF at varying BDF concentrations in RPMI using whole blood as a catalyst inside an incubator without stirring **a)** with a closed cap, and **b)** with an air balloon.

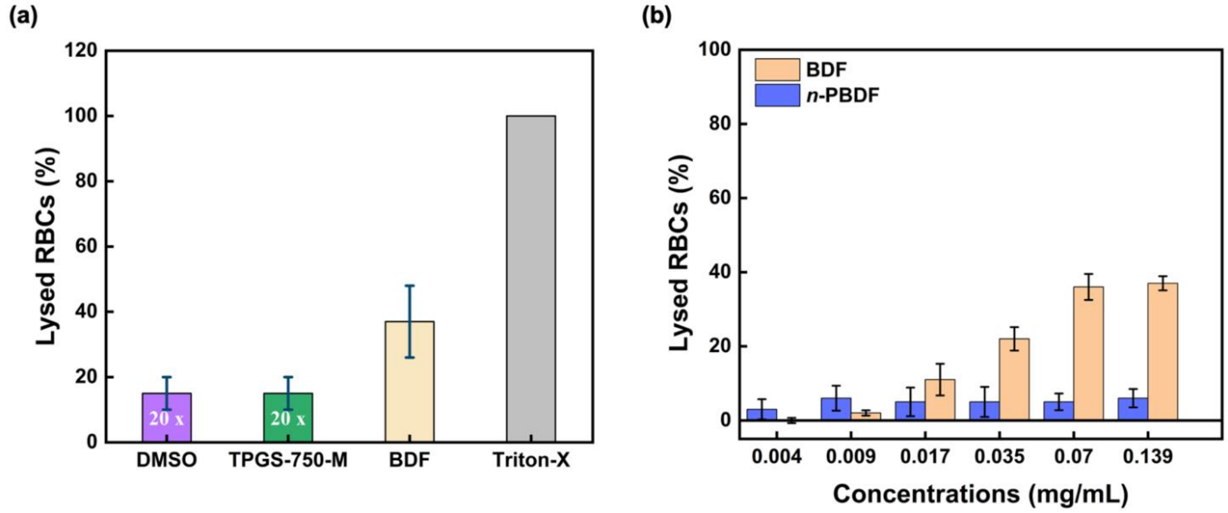

Figure S15. Hemolysis with **a)** Reaction components (5% v/v DMSO, 1% w/w TPGS, 0.2 mg/mL BDF), and **b)** BDF monomer and n-PBDF polymer

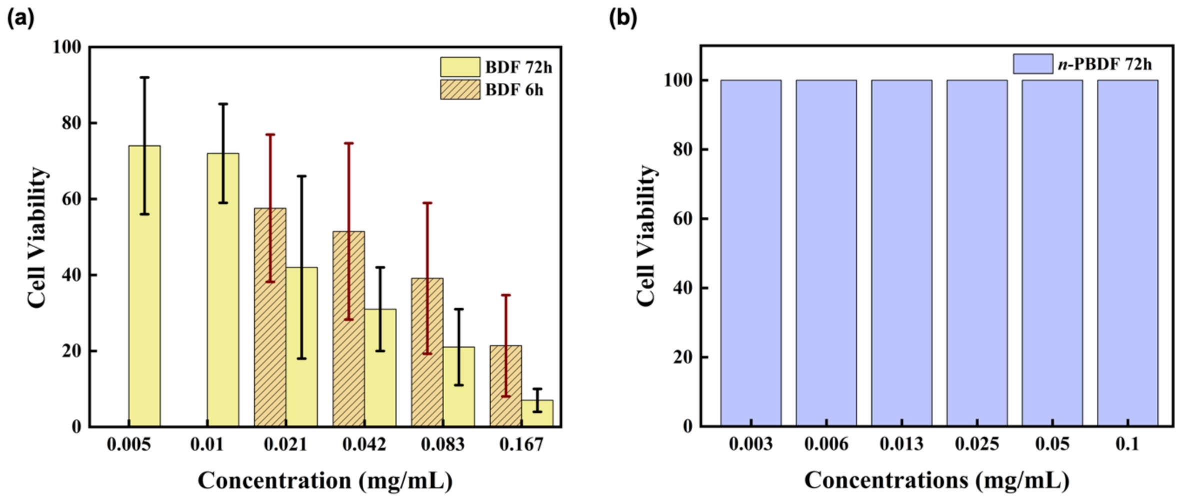

Figure S16. SRB cytotoxicity data of **a)** BDF monomer and **b)** n-PBDF polymer.

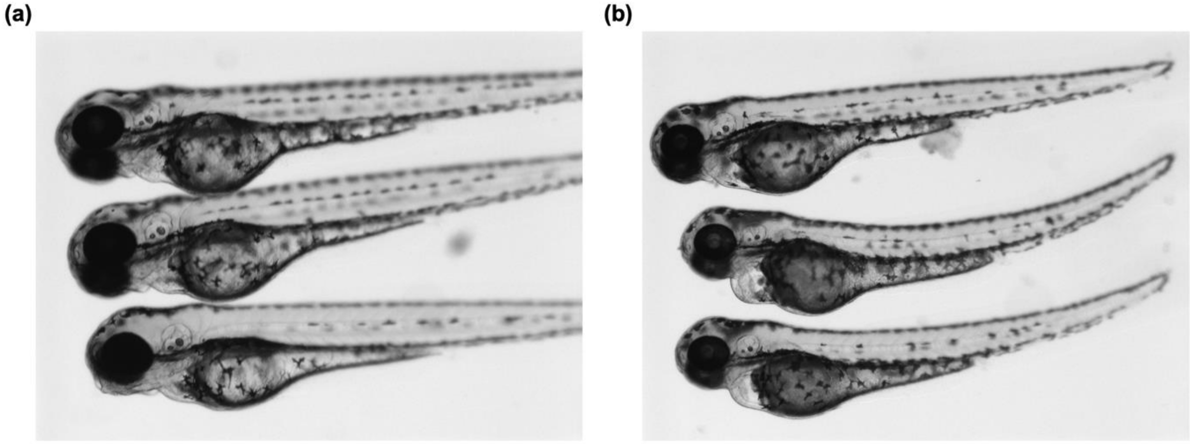

Figure S17. *In-vivo* formation of n-PBDF inside zebrafish embryo after 24h incubation at 34°C **a)** control, and **b)** 10mM BDF injection.

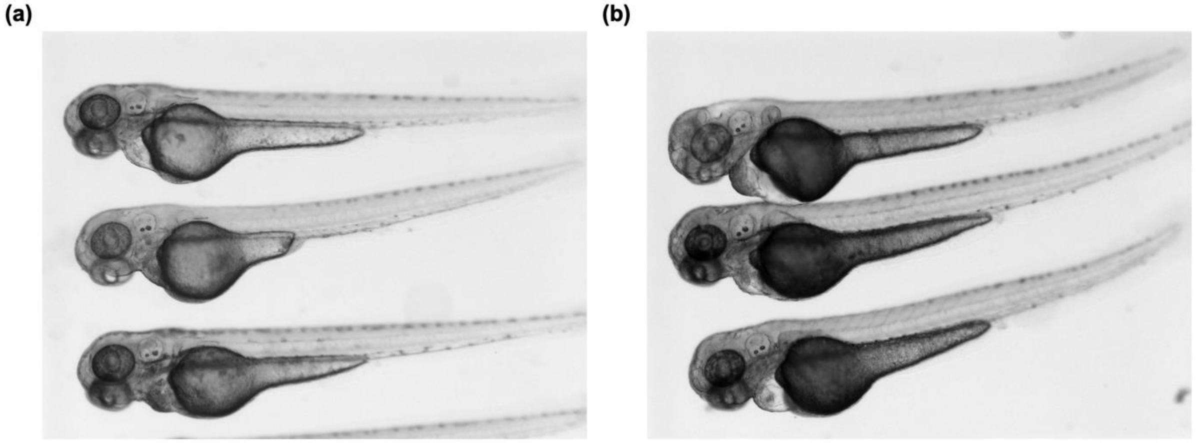

Figure S18. *In-vivo* formation of n-PBDF inside zebrafish embryo with PTU treatment after 24h incubation at 34°C **a)** control, and **b)** 10mM BDF injection.

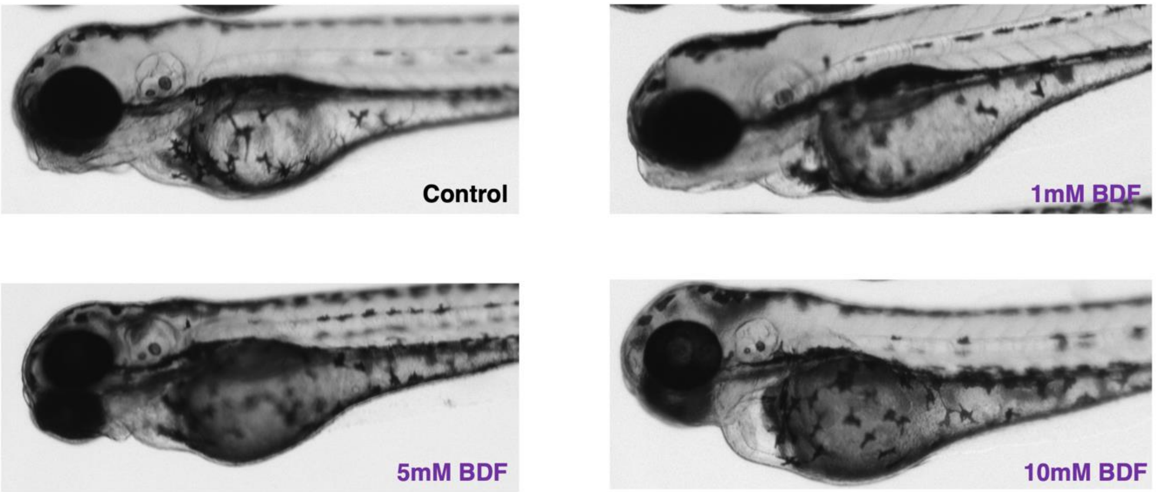

Figure S19. *In-vivo* formation of n-PBDF inside zebrafish embryo with varying BDF injection after 24h incubation at 34°C.

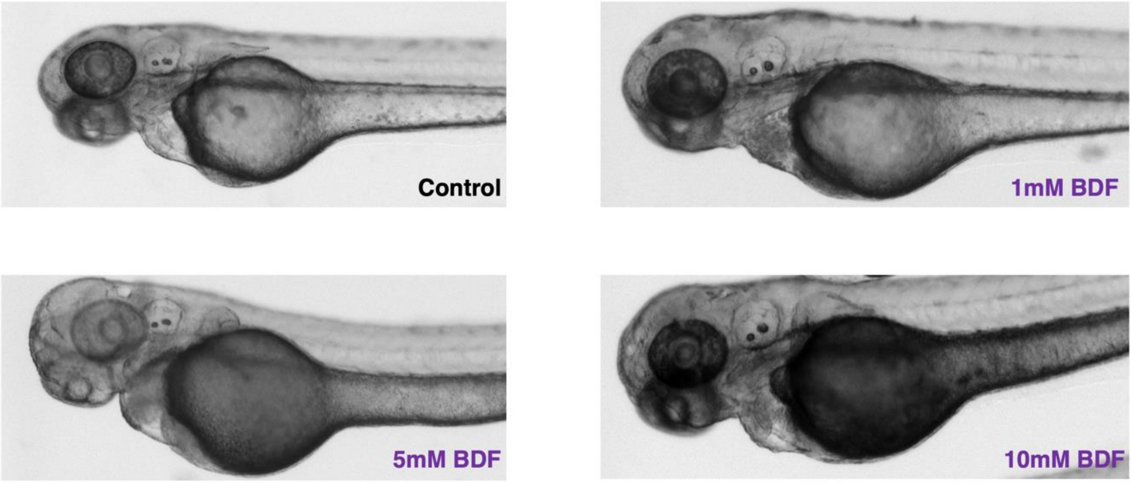

Figure S20. *In-vivo* formation of n-PBDF inside zebrafish embryo with varying BDF injection after 24h incubation at 34°C and with PTU treatment throughout the experiment.

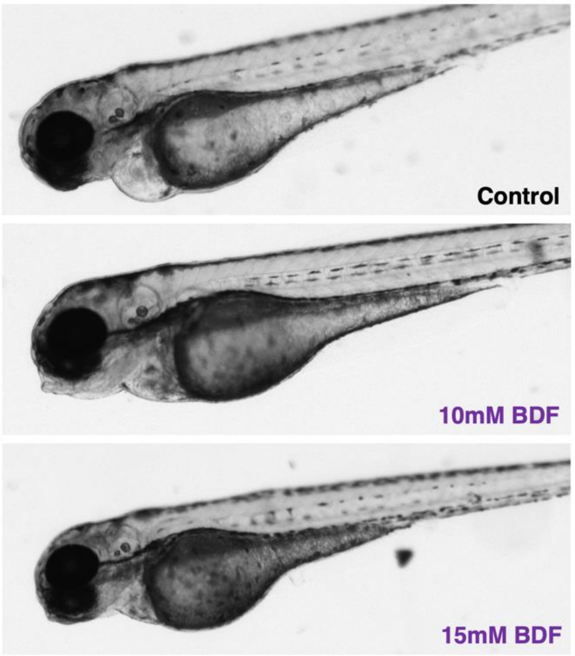

Figure S21. *In-vivo* formation of n-PBDF inside zebrafish embryo with varying BDF injection after 24h incubation at 34°C and with PTU treatment only until the BDF injection.

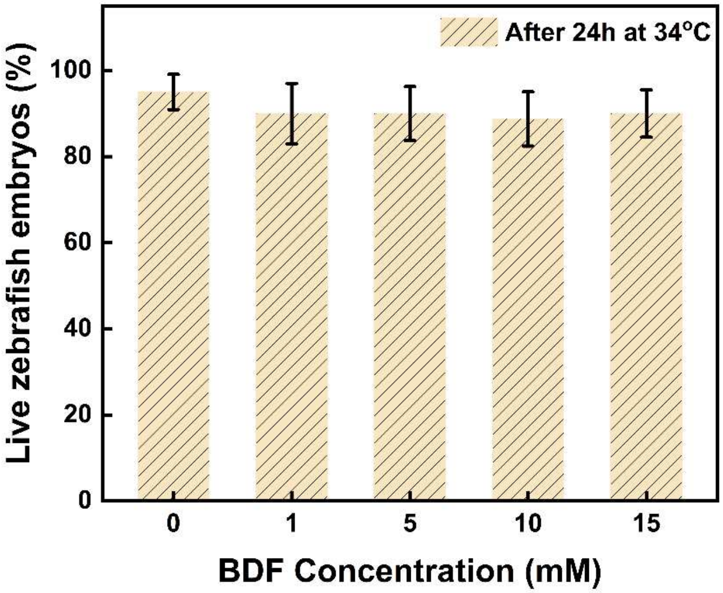

Figure S22. Live zebrafish embryos after 24h incubation at 34°C with different BDF injection concentrations.

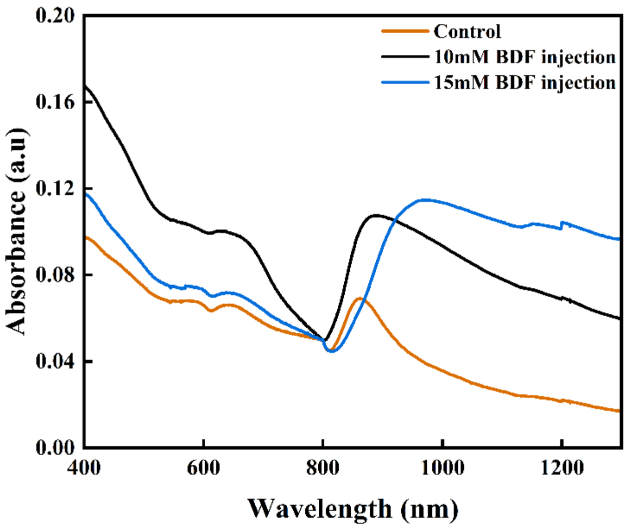

Figure S23. UV-Vis-NIR spectrum of zebrafish embryos after 24h incubation at 34°C with different BDF injection concentrations.

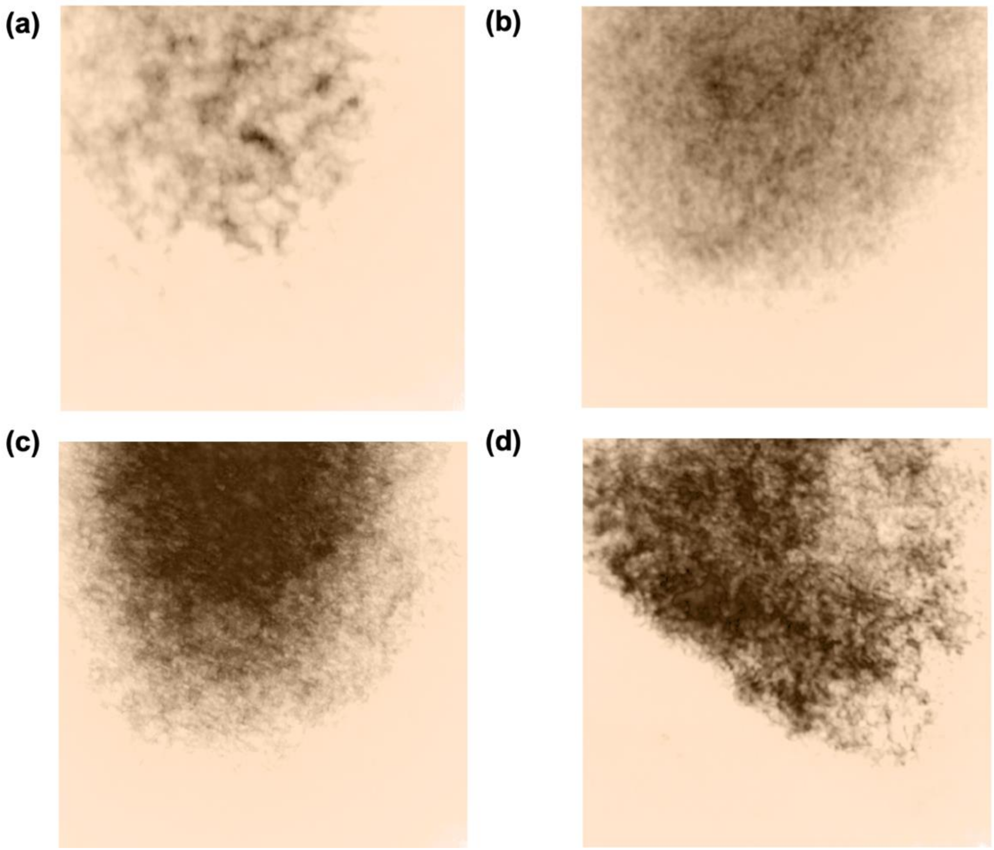

Figure S24. Absorption image of zebrafish embryo's yolk at 960 nm after 24h incubation at 34°C with different BDF injection concentrations, **a)** Control, **b)** 10mM BDF, **c)** 15mM BDF, **d)** 15mM BDF with PTU treatment.

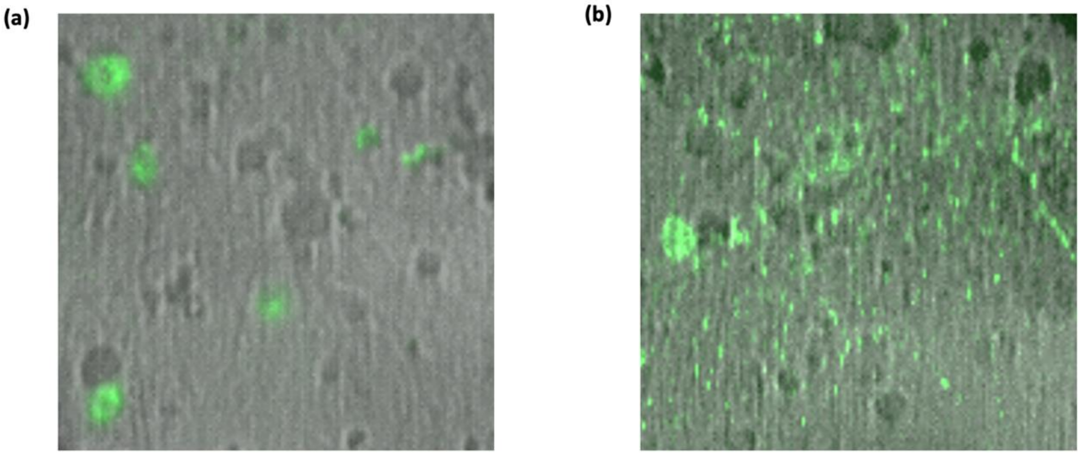

Figure S25. Transient absorption images of cultured neurons with *in cellulo* formed n-PBDF using Hb as the catalyst at 37 °C after an incubation period of **a)** 2 hours, and **b)** 16 hours.

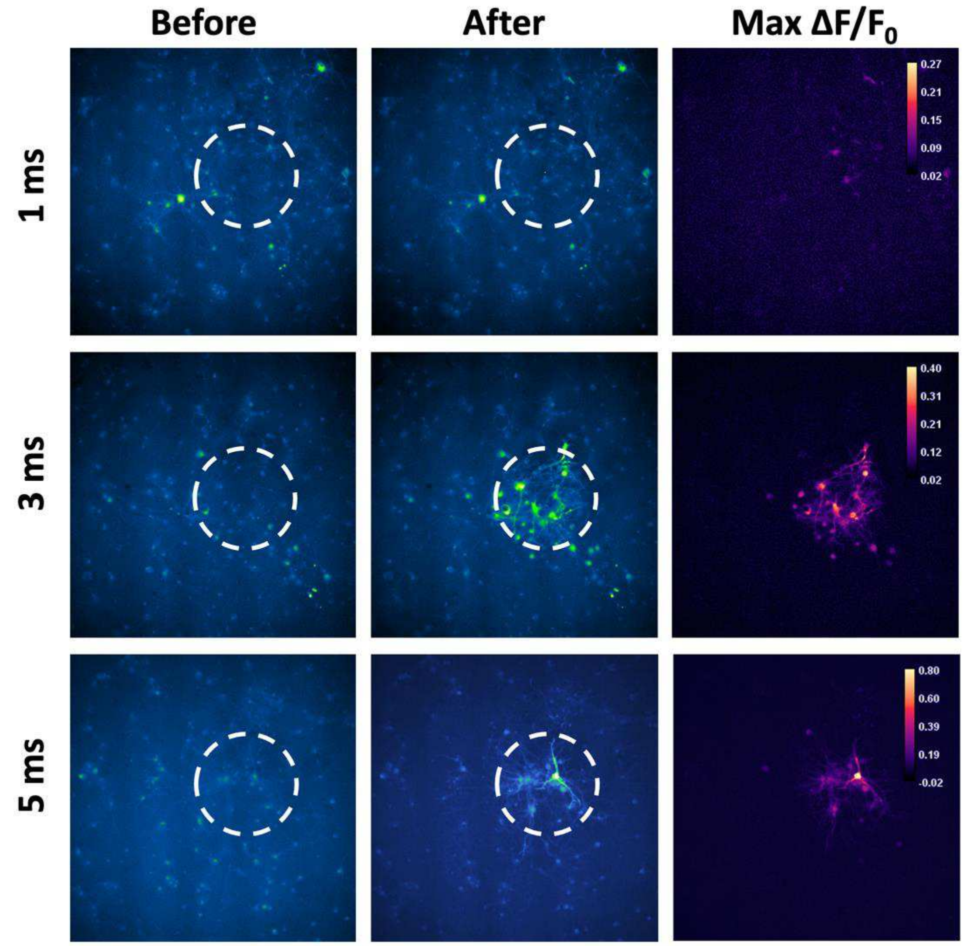

Figure S26. Fluorescence with Max $\Delta F/F_0$ images of Oregon Green labeled neurons upon a nanosecond 1030nm pulse laser with *in cellulo* formed n-PBDF at different exposure times.

References

1. Lei, T., Dou, J.-H., Cao, X.-Y., Wang, J.-Y. & Pei, J. Electron-Deficient Poly(p -phenylene vinylene) Provides Electron Mobility over 1 cm 2 V –1 s –1 under Ambient Conditions. *J. Am. Chem. Soc.* **135**, 12168–12171 (2013).
2. Ke, Z. *et al.* Highly Conductive and Solution-Processable n-Doped Transparent Organic Conductor. *J. Am. Chem. Soc.* **145**, 3706–3715 (2023).

Supplementary Files

This is a list of supplementary files associated with this preprint. Click to download.

- VideoS1.mov
- VideoS2.mov
